# One Health epidemic preparedness: Biosafety quality improvement training in Nigeria

**DOI:** 10.14202/ijoh.2023.10-14

**Published:** 2023-03-27

**Authors:** P. M. Davwar, D. P. Luka, D. F. Dami, D. D. Pam, C. T. Weldon, A. S. Brocard, S. Paessler, S. C. Weaver, N. Y. Shehu

**Affiliations:** 1. Department of Internal Medicine, Jos University Teaching Hospital, Plateau State, Nigeria;; 2. Department of Biotechnology, National Veterinary Research Institute, Plateau State, Nigeria;; 3. A. P. Leventis Ornithological Institute, University of Jos, Plateau State, Nigeria;; 4. Department of Zoology, University of Jos, Plateau State, Nigeria;; 5. Departments of Pathology, Microbiology & Immunology, University of Texas Medical Branch, Texas, USA;; 6. Biosafety Consulting, VA, USA.

**Keywords:** biosafety, One Health, pandemic, training, biological safety

## Abstract

**Background and Aim:**

One of the key components of the One Health approach to epidemic preparedness is raising awareness and increasing the knowledge of emerging infectious diseases, prevention, and risk reduction. However, related research can involve significant risks to biosafety and biosecurity. For this purpose, we organized a multidisciplinary biosafety hands-on workshop to inform and increase the knowledge of infectious diseases and risk mitigation. This study aimed to describe the process and outcome of a hands-on biosafety training program using a One Health approach across a multidisciplinary and multi-specialty group in Nigeria.

**Materials and Methods:**

A face-to-face hands-on training for 48 participants was organized by the West African Center for Emerging Infectious Diseases (WAC-EID) at the Jos University Teaching Hospital, serving as a lead institution for the Nigeria project site. Topics covered included (1) an overview of the WAC-EID research; (2) overview of infection prevention and control; (3) safety in animal handling and restraint, sample collection, and processing; (4) safety in field studies including rodent, bird and bat handling; (5) safety practices in the collection of mosquito and other arthropod vectors; (6) personal protective equipment training (disinfection, donning and doffing); and (7) safety in sample collection, labeling, and transportation. The program was executed using a mixed method of slide presentations, practical hands-on sessions, and video demonstrations. Pre- and post-course evaluation assessments and evaluation measures were used to assess training.

**Results:**

A total of 48 trainees participated in this training, with 12 (25%), 16 (33.3%), 14 (29.2%), 6 (12.5%) categorized as ornithology, entomology, mammalogy, and clinical interest groups, respectively. The pass rate for the pre-test was 29.4%, while for the post-test, it was 57.1%, or a 28% improvement. 88.6% of the trainees rated the training as relevant to them.

**Conclusion:**

Didactic and hands-on biosafety training is relevant in this era of zoonotic epidemics and pandemic preparedness. During this training program, there was a clear demonstration of knowledge transfer that can change the current practices of participants and improve the safety of infectious diseases research.

## Introduction

The “One Health” concept describes a situation where diverse sectors communicate and work to achieve change in public health outcomes. It represents the inseparability of the interaction between human, animal, and environmental health, creating a unified healthcare view [1].

The One Health approach can improve our understanding of the emergence and control of zoonotic disease. Zoonotic pathogens have proven to be the source of over 70% of emerging and re-emerging infectious diseases and spillover to human populations [2]. A case in point is the ongoing COVID-19 pandemic, which has caused the death of over 3 million people across the globe [3]. This leaves us with the constant fear of the unknown; what will be the next pandemic, and from where will it emerge?

If our interaction with wildlife and our environment is tilted toward benefits to humans alone and not a mutual benefit, then we will continue to expect spillover from animals to humans with the attendant devastating consequences of pandemics to our livelihood and economies [4].

The most recent epidemics of zoonotic diseases, namely, severe acute respiratory syndrome, H1N1 influenza, Middle East respiratory syndrome, and possibly COVID-19 which has a probable animal source, emphasize the need for us to be prepared for future interactions between animals, pathogens and humans. The severe economic and social consequences of zoonoses causing epidemics are evident because the world has become a global village with fast interconnectivity and ease of international trade. As humans continue to interact with wildlife and the environment, the risk of spillover increases. These biological threats can be enhanced through globalization and may spread, such as wildfires, resulting in pandemics. Hence, biosafety containment measures related to work on zoonotic pathogens are essential arsenal to One Health research. The potential of accidental contamination or release of very harmful biological agents into the community is also real and has occurred in the past. Thus, biosafety training and the availability of specialized laboratories for this important research is a necessity now more than ever before [5]. The need for constant training of the participants in the realm of One Health cannot be over-emphasized. Human medicine, veterinary sciences, and social and environmental sciences must all partner and be educated in a holistic way where each sector understands the interconnectivity between the three groups and their roles in preventing disease outbreaks whenever possible. Because of the complexity of the mechanisms leading to outbreaks of zoonotic diseases, preventing them requires synergistic action between multiple groups with complementary skills and knowledge.

One of the key components of the One Health approach is raising awareness in addition to increasing knowledge about emerging diseases, prevention, and risk reduction [6].

A second approach is expanding the scientific base on the inquiry into the complex nature of emerging diseases through surveillance and experimental research. To accomplish these goals safely, we organized a multidisciplinary didactic and hands-on biosafety workshop with the goal of raising awareness and increasing knowledge on emerging diseases and risk prevention.

This study aimed to describe the process and outcomes of a hands-on biosafety training program using the One Health approach across a multidisciplinary expert group in Nigeria.

## Materials and Methods

### Ethical approval and informed consent

This study was approved by the National Research Ethics Committee of Nigeria under protocol NHREC/01/01/2007– 30/11/2020 from Jos University Teaching Hospital.

### Training program design

**Figure-1:**
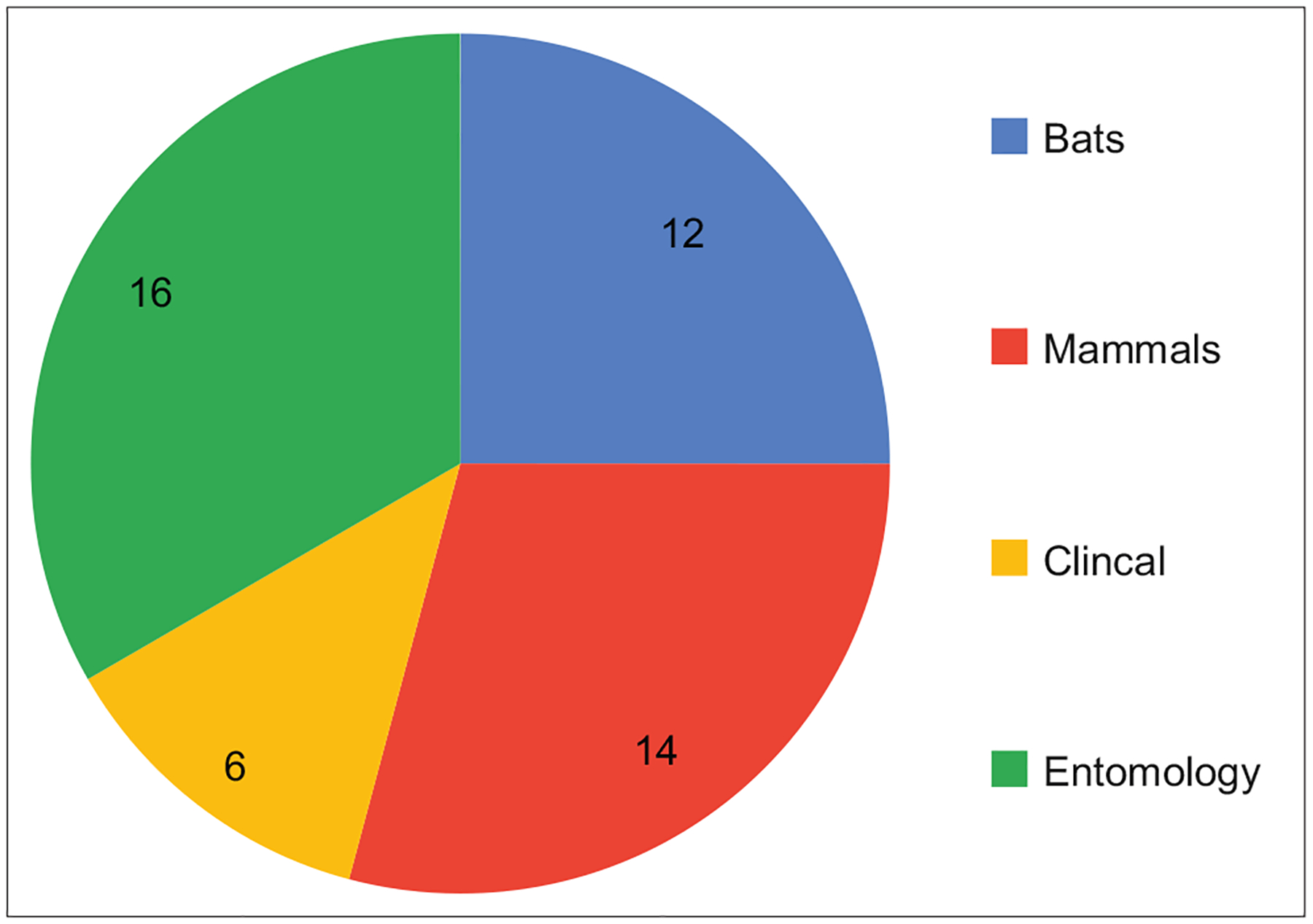
Pie chart showing distribution of the different specialty groups represented among participants.

A face-to-face hands-on training for 48 participants was organized by the West African Center for Emerging Infectious Diseases (WAC-EID) at the Jos University Teaching Hospital, serving as a lead institution for the Nigeria project site. Topics covered included (1) an overview of the WAC-EID research; (2) an overview of infections prevention and control; (3) safety in animal handling and restraint, sample collection, and processing; (4) safety in field studies including rodent, bird and bat handling; (5) safety practices in the collection of mosquito and other arthropod vectors; (6) personal protective equipment training (disinfection, donning and doffing); and (7) safety in sample collection, labeling, and transportation. There were 8 faculty members drawn from the University of Jos, Jos University Teaching Hospital, National Veterinary Research Institute Vom, and AP Leventis Ornithological Institute. They included virologists, infectious diseases physicians, medical microbiologists, ornithologists, and medical entomologists, as shown in Figure-1. The program was executed using a mixed method of slide presentations, practical hands-on sessions, and video demonstrations with pre- and post-course evaluation assessments, and evaluation measures were used to assess training.

### Study period and location

The hands-on biosafety training was carried out on April 9, 2021. The site of the training was the multipurpose hall of the Jos University Teaching Hospital, with a capacity to seat 400 people with enough space for breakout sessions.

### Preparation for the training event

Several training meetings took place over a period of 4 weeks among the organizers of the workshop, including both in-person and virtual meetings. A list of training content was generated during those meetings and trainers were identified based on nominations from the organizers and expertise in diverse fields. They were contacted through emails and phone calls.

### Overview of the biosafety training

This was a 7-h training program with a 15 min break and a 30 min lunch break. The sessions included didactic teaching, hands-on training, and practical sessions. The course content included the following: (1) an overview of the West African Center for Emerging Infectious Disease (WAC-EID) research; (2) an overview of infections prevention and control; (3) safety in animal handling and restraint, sample collection, and processing; (4) safety in field studies including rodent, bird, and bat handling; (5) safety practices in the collection of mosquito and other arthropod vectors; (6) personal protective equipment training (disinfection, donning, and doffing); (7) safety in sample collection, labeling; and (8) transportation, followed by breakout sessions with Principal Investigators. Items 6, 7, and 8 were hands sessions; the others were didactic sessions.

### Participants

The participants were selected from the core groups of the WAC-EID. There were 48 participants from the ornithology, entomology, mammalogy, and clinical research groups.

### Data collection

Pre- and post-test evaluations were collected on paper forms and data were entered in excel spread-sheets for comparison of pre and post-test scores.

Questions were drawn from all topics covered during the training with each topic having an equal number of questions. The questions were designed in multiple choice format with a single best answer and short answer questions.

The training evaluation feedback also comprised a 5-point Likert scale ranging from 1 for completely agree to 5 for completely disagree. The themes that were measured in the evaluation survey pertaining to the workshop logistics were: Format, venue and room arrangement, planning communication, travel logistics, time management, productivity, and length of training.

Elements of the workshop related to content that were evaluated consisted of: Clarity of presentations, relevance of training and material, organization of content, clarity of instruction, training effectiveness, and active participation.

### Statistical analysis

Data were captured and stored in an Excel spreadsheet and then analyzed using SPSS version 20 (IBM Corp., Armonk NY, USA). Frequency tables were generated and descriptive statistics, including proportions, percent, mean (standard deviation), and median (Interquartile range) were reported for categorical and continuous variables. The Likert scores of training evaluation were analyzed using mean scores.

## Results

**Figure-2:**
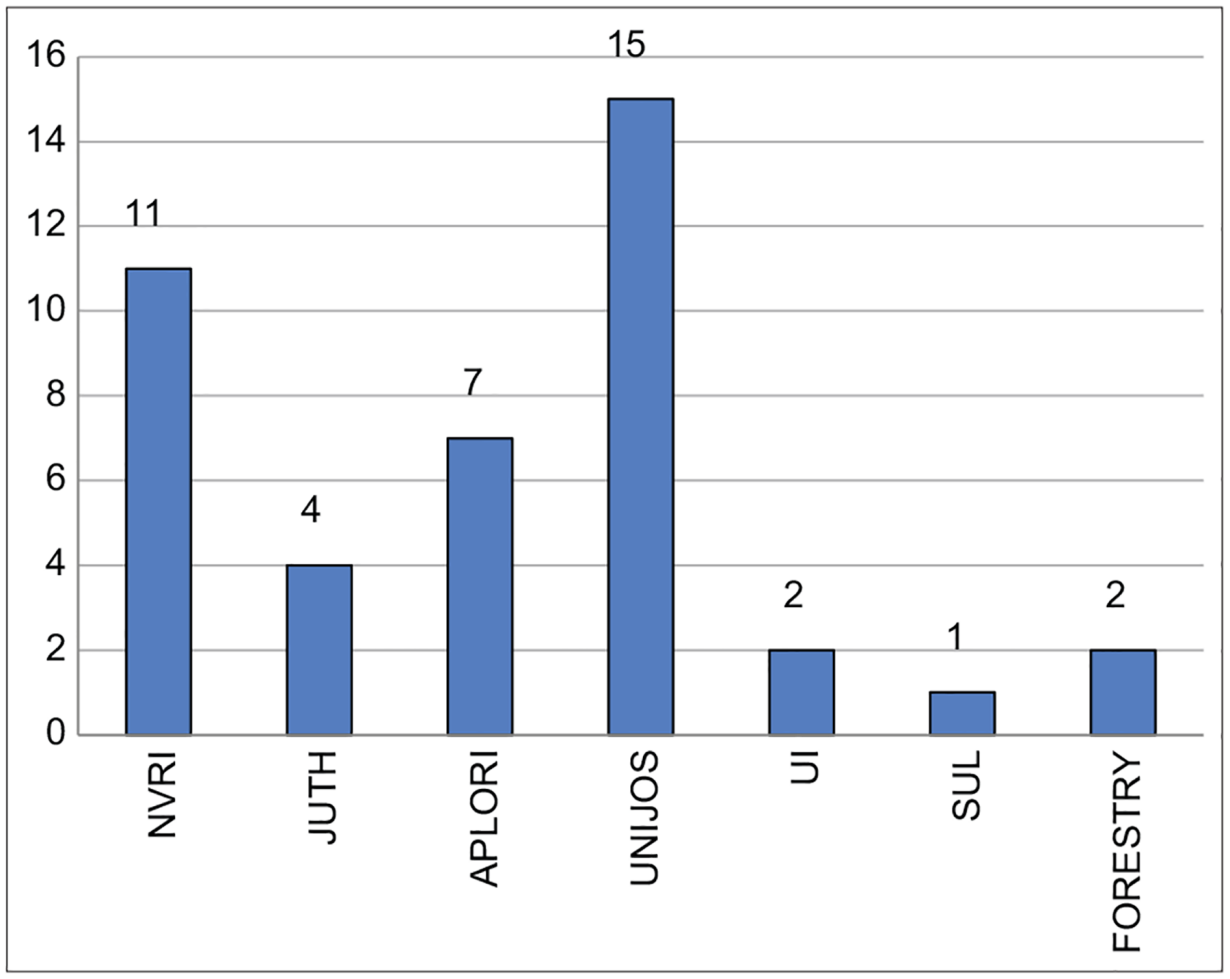
Bar Chart showing different institutions represented. NVRI: National Veterinary Research Institute, JUTH: Jos University Teaching Hospital, APLORI: AP Leventis Ornithological institute, UI: University of Ibadan, SUL: Lafia State University, Forestry: School of Forestry Jos, UNIJOS: University of Jos.

There was a total of 48 participants in this training; 12 (25%) were from ornithology, 16 (33.3%) from entomology, 14 (29.2%) from mammalogy, and 6 (12.5%) from clinical sections. Figure-2 shows the distribution of the participants based on the different institutions present.

**Figure-3:**
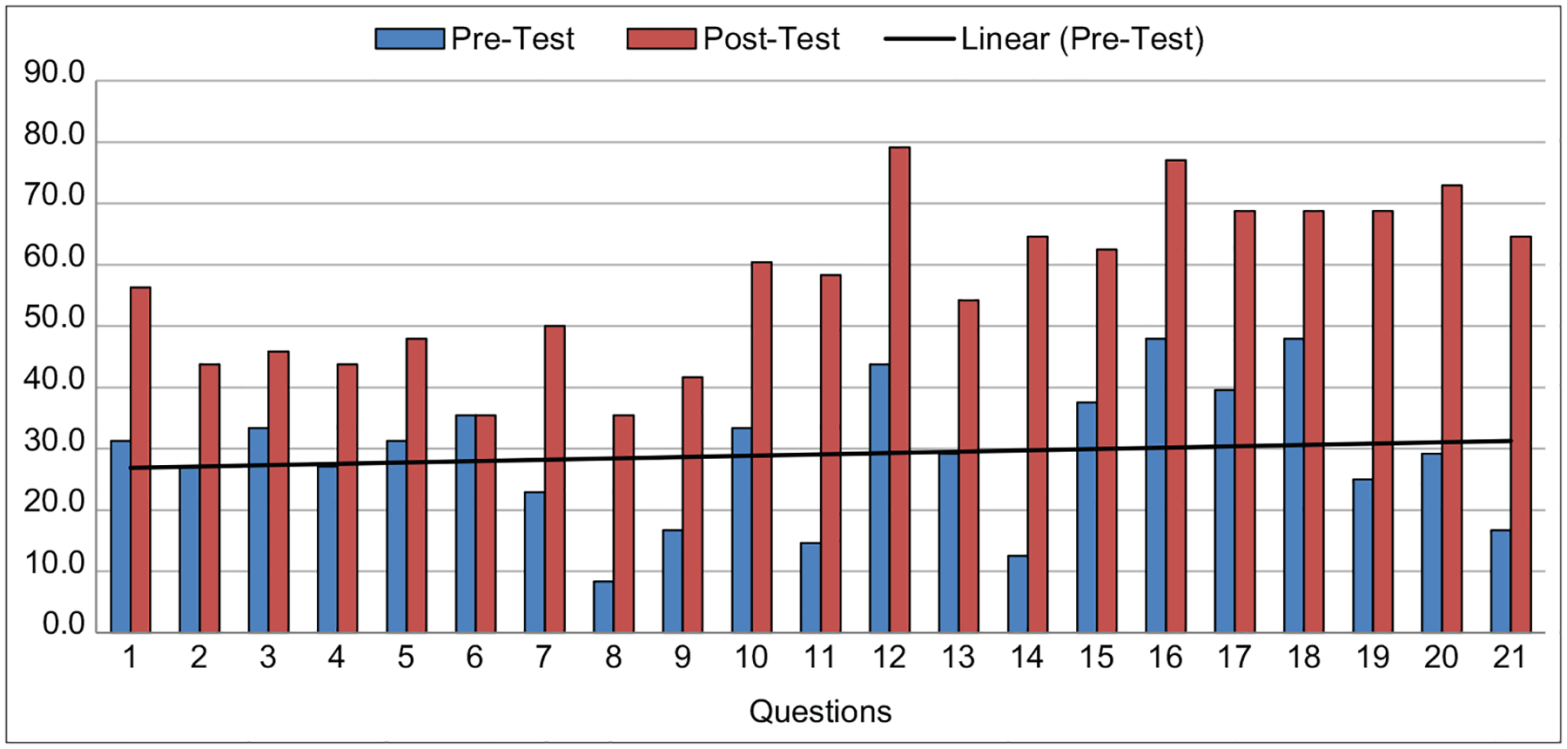
Percentage score for pre- and post- tests for each question.

**Figure-4:**
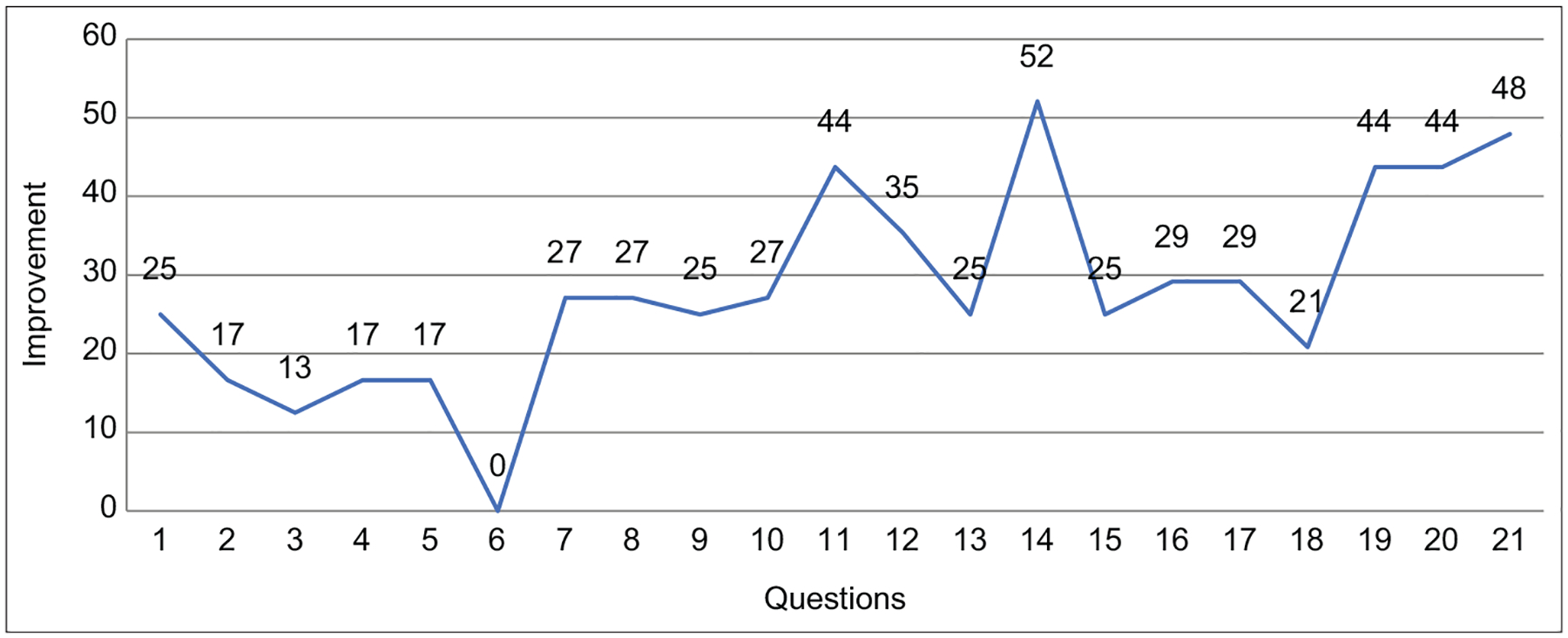
Line graph showing the percentage improvement for each question from the pre- and post-test.

The average percentages of correct scores for the pre- and post-test were 29.4% and 57.1%, respectively or a 28.1% improvement (Figures-3 and 4). Overall, there was a statically significant difference between the pre- and post-test scores of the study participants.

Overall, 88.6% of the trainees found the training valuable to them, while 82.6% said what was discussed during the training was relevant to them; 71.4% thought the explanation was clear and easy to understand and that the presentation was effective; 62% thought that the material provided was useful to them and 68% thought that the content was well organized. Only 50% of the participants thought that the length of training was sufficient.

## Discussion

We describe a cross-sectional observational study that was carried out to evaluate didactic and hands-on biosafety training at the Jos University Teaching hospital related to surveillance and research activities. The training was based on the One Health model, where participants were drawn from a multidisciplinary team that consisted of medical clinicians, veterinary specialists, zoologists, and ecologists. This breadth of participants ensured coverage of all the components of the One Health approach to healthcare. Training and education in both human and veterinary forms of medicine, where the health of both animals and humans become important, are considered to be an important approach to combat epidemics. Similar training has been implemented in Makerere University [7]. This was, however, done among undergraduate students and only in one institution, whereas our training involved multiple organizations and is for practitioners, lecturers, and researchers. The ability to encompass multi-sectoral professions in these types of training is needed in the early phase of creating awareness for One Health approach. The participants rated the training as relevant to their research and practices. There was a significant improvement in the pre- and post-test scores overall for the study participants and an average improvement of about 25% for each question. Overall, there was a measurable knowledge transfer during this training on biosafety and epidemic preparedness.

The importance of the One Health approach, particularly when it comes to knowledge integration, cannot be over-emphasized as this has become apparent during the COVID-19 pandemic [7, 8]. To prevent such future recurrences of such diseases in epidemic proportions, such biosafety training becomes a necessity where clear learning is demonstrated and change in practice achieved. Furthermore, this biosafety training included participant feedback on the relevance of the training in improving their current practices with respect to infection prevention and control and sample labeling and transport among others. A similar study carried out in Uganda on needs assessment for biosafety training among healthcare workers was supported by over 90% of the participants [9]. That study highlighted the perceived benefits of biosafety training to include individual capacity development, community, national, and global health issues.

To achieve preparedness for pandemics, knowledge and capacity development are key components [10, 11]. Our training provided both knowledge and capacity development as demonstrated by the results of our pre- and post-evaluation. The blend of both medical clinicians and veterinarians in this training meets one of the key components of the One Health concept. Although the veterinary community is in tune with this concept, the medical community in Nigeria and many other regions have not fully embraced this component [12]. Thus, having experts from both specialties participate in hands-on training can serve as a model for a better understanding of the role each plays in matters related to biosafety and pandemic preparation. Future training should also include a focus on animal welfare for One Health approach to be complete.

## Conclusion

The global community is constantly under threats of zoonotic emergence. We are constantly threatened by the appearance of new diseases with epidemic potential. This is fueled by human encroachment into the spaces and habitats of wild animals, giving ample opportunity for an interface between humans and animals that did not exist before and with it the opportunity for spread of zoonotic diseases. These interactions will continue to happen. Hence, the need for concerted efforts to ensure that these activities do not lead to spillover infections and, if they do occur, that we are prepared to respond rapidly and precisely; this will require a timely and an organized response. Preparedness and training using a One Health approach are essential to anticipating, preventing, mitigating, and containing emerging infectious diseases.

## Data Availability

Supplementary data can be available from the corresponding author upon a reasonable request.
